# Mesenchymal stem cells derived extracellular vesicles for chronic kidney disease: pleiotropic mechanisms of actions of a versatile therapy

**DOI:** 10.3389/fbioe.2025.1612193

**Published:** 2025-06-13

**Authors:** Elena Ceccotti, Marco Quaglia, Giovanni Camussi, Stefania Bruno

**Affiliations:** ^1^ Department of Medical Sciences, University of Torino, Turin, Italy; ^2^ Department of Translational Medicine, Università del Piemonte Orientale (UPO), Novara, Italy; ^3^ Nephrology and Dialysis Unit, SS. Antonio e Biagio e Cesare Arrigo University Hospital, Alessandria, Italy

**Keywords:** chronic renal damage, cell-free therapy, exosomes, fibrosis, murine models

## Abstract

Chronic kidney disease (CKD) has increasingly become a major health concern worldwide, globally affecting 10%–15% of adults, with significant implications for morbidity and mortality. This progressive condition can potentially evolve into end-stage renal disease (ESRD), requiring dialysis or renal transplant. However, the heaviest impact of CKD is due to an associated increased cardiovascular risk, due to frequently coexisting hypertension and diabetes and non-traditional risk factors, including accumulation of atherogenic toxins, alteration of calcium-phosphate balance, oxidative stress and chronic microinflammation. Mesenchymal stem cells (MSCs) have been proposed as a therapy for CKD due to their immunomodulating and tissue repairing properties. It has been proposed that extracellular vesicles (EVs) may mediate the therapeutic effects of the cells of origin and MSC-EVs have shown promise as treatment of different aspects of CKD in experimental settings. Their anti-fibrotic and anti-apoptotic properties may inhibit progression of CKD and promote healing of tubular and glomerular damage. MSC-EVs can prevent epithelial-mesenchymal transition, a key mechanism of evolution of acute kidney injury towards CKD. These actions may inhibit development of interstitial fibrosis and accumulation of the extracellular matrix components (ECM), key lesions which promote the progression of CKD. Furthermore, MSC-EVs also exert anti-inflammatory and anti-oxidant properties which may reduce vascular damage and cardiovascular risk associated with CKD. For example, Human Liver Stem Cell (HLSC)-derived EVs (HLSC-EVs) can reverse renal and cardiac alterations. As shown in a murine model of partial nephrectomy, HLSC-EVs shuttled proteases with ECM remodeling activity, lending support to the possibility of a simultaneous cardio-nephroprotective effect. Adipose, umbilical cord and inducible- MSCs are other possible sources of EVs potentially applicable to obtain reparative processes in CKD and ESRD. Overall, building experimental evidence suggests that MSC-EVs derived from different sources are a promising therapeutic tool to prevent development and progression of CKD and to reduce related cardiovascular risk. The strength of this therapy lies in its multi-level and pleiotropic actions which appear to interfere with many key etiopathogenetic mechanisms of CKD. Interesting future perspective is a combined therapy associating MSC-EVs with drugs to achieve synergistic effects and recent finding indicate the feasibility of this approach.

## 1 Introduction

Chronic kidney disease (CKD) is defined as the presence of structural abnormalities or a progressive decline in kidney function due to renal damage, lasting at least 3 months and is categorized according to the level of glomerular filtration rate (GFR) and albuminuria. It globally affects 10%–15% of adults, with significant implications for morbidity and mortality and a trend to an increase over the last decades, which makes it an important health concern worldwide (Qin et al., 2024). By 2040, CKD is estimated to become the fifth cause of death globally, representing one of the largest expected rises among major causes of death (GBD, 2021 Forecasting Collaborators, 2024).

This progressive condition can potentially evolve into end-stage renal disease (ESRD) if not adequately treated, requiring dialysis or renal transplant. However, the heaviest impact of CKD, even at initial stages, is due to an associated increased cardiovascular risk (Caturano et al., 2024). This is partly mediated by coexisting hypertension, cardiovascular disease and diabetes and partly due to a wide range of “non-traditional” risk factors, including accumulation of atherogenic toxins, alteration of calcium-phosphate balance and vascular calcifications, oxidative stress, chronic microinflammation and impaired energetic metabolism (Ferdinand, 2024). Another typical feature of CKD is a high incidence of infections due to an altered innate and adaptive immune response; cardiovascular disease and infections together account for up to 70% of deaths among CKD patients (Syed-Ahmed and Narayanan, 2019).

Current treatments of CKD include, in addition to therapy of primary renal disorder whenever possible, several classes of drugs which have proven effective in reducing proteinuria and cardiovascular risk and slowing progression towards ESRD. These include renin-angiotensin system (RAS)-blockers, sodium-glucose cotransporter 2 inhibitors (SGLT-2i) as a first-line, whereas non-steroidal mineralocorticoid receptor antagonists and glucagon-like peptide 1 receptor agonists (GLP-1RA) can be added for diabetic patients to further strengthen nephro-cardioprotection (Kidney Disease: Improving Global Outcomes CKD Work Group, 2024). While association of these classes of drugs can significantly modify the trajectory of progression of renal damage, current management of CKD is still challenging and new therapies are needed (Evans et al., 2022). Mesenchymal stem cell (MSC) and related extracellular vesicles (EVs) may represent a new tool to treat different aspects associated with CKD, from cardiovascular risk to progression of chronic renal damage, targeting underlying mechanisms (Hickson et al., 2016). Their immunomodulating and tissue-repairing properties have potential beneficial effects on different key biological processes associated with CKD. The combined therapy of MSC-EVs and drugs open a new perspective in treatment of CKD patients and this aspect will be explored in this review.

## 2 Main biological processes associated with CKD

**FIGURE 1:**
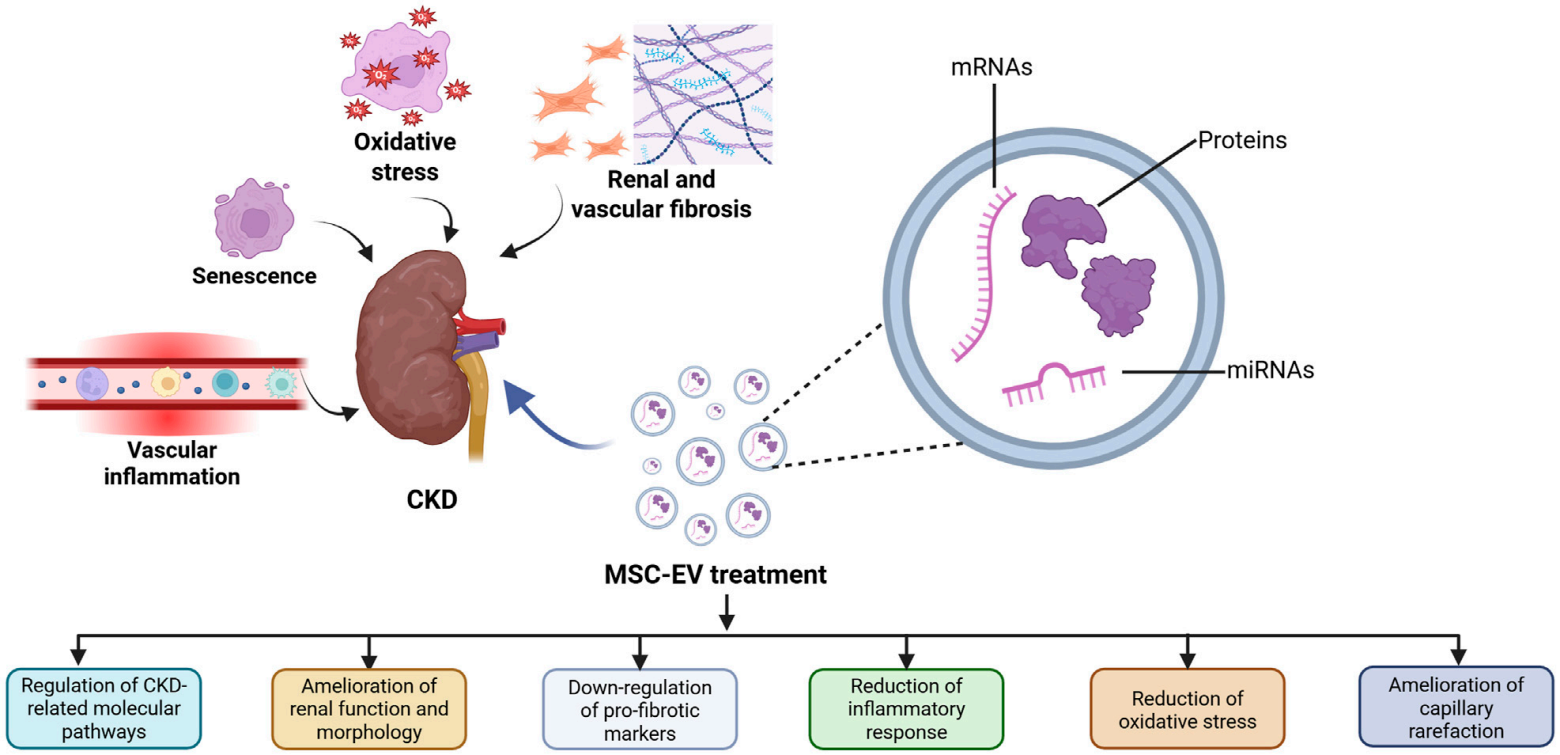
Therapeutic effects of MSC-EVs in CKD. Several biological processes characterize CKD development and MSC-EVs can counteract these processes by modulating different aspects implicated in CKD onset.

Several intertwined biological processes characterize CKD and determine typical clinical features, such as predisposition to cardiovascular disease, frailty and a tendency to develop a progressive course ultimately leading to accelerated renal and vascular fibrosis and aging (Wang C. et al., 2023). Interestingly, MSC-EVs can interfere with these different processes (Figure 1).

### 2.1 Renal fibrosis

Renal fibrogenesis is a complex process mediated by a wide range of mechanisms, including different types of cellular death (apoptosis, necroptosis and ferroptosis), cell cycle arrest, mitochondrial dysfunction, innate and adaptive immune reactions. Transition of interstitial fibroblasts into myoblasts and production of extracellular matrix (ECM) play a key role in this process, along with epithelial–mesenchymal transition (EMT) and endothelial to-mesenchymal transition (Frąk et al., 2024). This process is mediated by crucial pathways activated in response to inflammatory stimuli, such as transforming growth factor beta 1 (TGFβ) and Wnt/β-catenin. EMT determines loss of tubular function and impairment of regenerative tubular capacity due to induction of cell cycle arrest in G2 phase. In response to injury, tubular epithelial cells undergo functional changes and acquire properties similar to inflammatory and fibrogenic cells, such as the releasing cytokines and other mediators that trigger interstitial inflammation and fibrosis (Liu et al., 2018). This alteration of renal tubular cells becomes a driving force for CKD progression and mediates the evolution of acute kidney injury (AKI) towards CKD (AKI-to-CKD transition) through “maladaptive repair” (Neyra and Chawla, 2021; Lameire et al., 2021). The fibrotic process then develops through an interplay between injured tubular cell and non-parenchymal cell lineages, such as immune and mesenchymal cells and is associated with tubule atrophy, chronic interstitial inflammation, glomerulosclerosis and vascular rarefaction (Huang et al., 2023). Of interest, kidney fibrosis is largely modulated by epigenetic changes, mediated by specific microRNAs (miRNAs), and this aspect opens the possibility for a therapeutic manipulation of these mechanisms to inhibit or revert fibrosis. For example, miR-122-5p promotes renal fibrosis and inflammation in hypertensive rats, its pharmacological inhibition has a potential therapeutic significance (Liu et al., 2022).

### 2.2 Microinflammation and oxidative stress

Chronic microinflammation and oxidative stress are major contributors to pathophysiological and clinical features of CKD, especially at advanced stages, and exacerbate one another in a vicious circle (Cobo et al., 2018). Elevated circulating levels of pro-inflammatory cytokines such as tumor necrosis factor alpha (TNF-α), interleukin 6 (IL-6) and IL1β are found in CKD and can activate key-enzymes involved in radical oxygen species (ROS) production, such as nicotinamide adenine dinucleotide phosphate (NADPH) oxidase, xanthine oxidase and inducible NO synthase. The imbalance between increased ROS production and antioxidant defense can result in oxidative stress and damage lipids, proteins and DNA within the cell and in surrounding tissues, further promoting inflammation. For example, oxidative stress triggers activation of nuclear factor-kappa B (NF-kB), amplifying the inflammatory cascade (Frąk et al., 2024).

Mitochondrial dysfunction, caused by uremic toxins and characterized by impaired mitochondrial biogenesis and ATP generation, is an important contributor to oxidative stress in CKD causing leakage of electrons and generation of superoxide radicals (Srivastava et al., 2023). In physiological conditions, mitochondria produce low levels of ROS, which are thought to impact cell signaling processes such as hypoxia detection. However, these organelles become a crucial source of high levels of ROS leading to cellular damage and death under different pathophysiological conditions (Podkowińska and Formanowicz, 2020). Moreover, mitochondrial DNA (mtDNA) can interact with endoplasmic reticulum molecules, and it directly triggers tubulo-inflammatory responses and promotes the progression of CKD (Mao et al., 2022).

Within the kidneys, superoxide radicals are important promoters of oxidative stress mainly produced by NADPH oxidase (Ling and Kuo, 2018). Recent studies suggested the role of TGFβ/Smad signaling in ROS production. Indeed, TGFβ both induces NADPH oxidase activity, stimulating different molecular pathways such as Smad2/3 and NF-κB, and suppresses antioxidants, such as GSH, contributing to oxidative stress within renal tissue (Srivastava et al., 2023).

### 2.3 Cellular senescence

CKD can be considered as a clinical model of premature aging due to accelerated cellular senescence, which involves tubular cells, fibroblasts, endothelial cells and podocytes, both in patients and in animal models (Yang et al., 2025). Cellular senescence is characterized by activation of the DNA-damage-response, a dysfunctional antioxidant nuclear factor erythroid 2 related factor 2 (Nrf/2) pathway and downregulation of nephroprotective factors such as Klotho (Marquez-Exposito et al., 2022). Klotho protein has anti-inflammatory and antiaging effects and appears to protect mitochondrial function (Prud’homme and Wang, 2024). Interestingly, circulating soluble Klotho levels correlate directly with insulin resistance and inversely with protein energy wasting in uremic patients (Zhou et al., 2025). Moreover, Klotho supplementation determined a marked reduction of fibrosis index and renal expression of collagen I and TGFβ, along with a reduction of proteinuria in a rat model of remnant kidney (Takenaka et al., 2023).

Nrf/2/Heme Oxygenase-1 signaling axis provides multi-organ protection against oxidative stress and plays a pivotal role in mediating anti-inflammatory and anti-apoptotic responses; the impairment of this system is involved in premature vascular aging and CKD-related oxidative stress (Tanriover et al., 2023; Chaudhary et al., 2023).

Mechanisms of senescence also play an important role in the specific setting of AKI-CKD progression. Tubular cell senescence promotes profibrotic phenotype transformation and maladaptive kidney repair after cisplatin nephrotoxicity, favoring evolution towards CKD (Li et al., 2023). Senescence is closely intertwined with oxidative stress and low-grade chronic inflammation. The complex of these processes has been referred to as “inflammaging” and is the main underlying mechanism of vascular calcification and atherosclerotic damage in CKD (Ho et al., 2025). In addition to activation of innate immunity, CKD is also characterized by an abnormal adaptive response (Tang et al., 2024). Low-grade persistent inflammation and cellular senescence of kidney and vascular system often develop in parallel and are progressively amplified by age, contributing to the unique severity of “inflammaging” in CKD (Yamamoto and Isaka, 2024) (Figure 1).

## 3 Mesenchymal stem cells and their extracellular vesicles

MSCs are multipotent, non-hematopoietic stem cells and, according to the International Society for Cell and Gene Therapy (ISCT), they should be capable of self-renewal and to differentiate into three lineages (osteogenic, adipogenic, and chondrogenic) (Viswanathan et al., 2019). Another important aspect to define MSCs is their phenotype which includes the expression of cell-surface markers as CD105, CD73, and CD90, and is negative for the hematopoietic and endothelial markers, as CD45, CD34, CD14, CD19 (Wang et al., 2024).

MSCs can be isolated from various tissues, such as Wharton’s jelly of the umbilical cord (UC) and bone marrow (BM), and they show regenerative and immunomodulatory properties in the fibrotic organs. Indeed, in the damaged tissues MSCs regulate the functions of immune cells, such as T and B lymphocytes and antigen-presenting cells, influencing antigen recognition and elimination (Qin et al., 2023; Wang et al., 2024). Considering these aspects, in the last years MSCs have presented a promising strategy for treating renal damage, particularly in those patients where the maladaptive repair after AKI predisposes to CKD development. Based on recent studies, it has been highlighted that MSC-based therapies could improve kidney inflammation by regulating both the differentiation of dendritic cells and mTOR pathway (Zhang et al., 2020; Luo et al., 2021). Moreover, MSC biological cargo includes anti-fibrotic miRNAs and proteins that can be released in the target cell modulating renal fibrosis (Joo et al., 2020).

The regenerative and immunomodulatory properties of MSCs are mediated by the secretion of paracrine factors including the extracellular vehicles (EVs), involved in cell-cell communications both in physiological and pathological conditions. They are lipid-bilayer enclosed nanoparticles with a biological cargo that reflects the cell of origin and includes cytokines, growth factors, mRNAs and non-coding RNA (Jafarinia et al., 2020). MSC-derived EVs express typical cell-surface tetraspanins, such as CD9, CD63 and CD81, and represent a promising safe and biocompatible cell-free therapy with low immunogenicity and not able to self-replicate (Kou et al., 2022). Thanks to the lipid membrane, their structure and cargo can be preserved for prolonged periods, and they can be more easily internalized into target cells, thus improving the interest in developing EV-based therapies to treat renal diseases. Recent experimental studies highlighted the ability of MSC-EVs to modulate key molecular pathways involved in CKD development and progression, such as oxidative stress, inflammation, angiogenesis and fibrosis (Nargesi et al., 2017). Different animal models of CKD, including ischemia-reperfusion injury (IRI), diabetic and hypertensive nephropathy, unilateral ureteral obstruction (UUO) and partial nephrectomy, have been developed to investigate the renoprotective properties of MSC-EVs and the contribution of their biological cargo to regulate the main pathways involved in the progression of renal damage (Eirin and Lerman, 2021).

### 3.1 Application of MSC-Derived EVs in CKD

The first evidence that MSC-EVs could have a beneficial effect on the progression of CKD was provided by the administration of a single dose of human BM-MSC-EVs immediately after renal IRI in rats (Gatti et al., 2011). In IRI-rats treated with a single-dose of BM-MSC-EVs and sacrificed 6 months after, an amelioration of renal function and morphology was observed with a reduction of signs of CKD development, such as interstitial fibrosis, lymphocyte infiltrates, tubular atrophy and cystic formations. The therapeutical effect of a single BM-MSC-EV administration was also confirmed in a murine model of UUO-induced CKD. Histological analyses demonstrated relatively well-preserved renal architecture and significant reduction of histological features of renal injury in mice treated with BM-MSC-EVs (He et al., 2015). Moreover, multiple administrations of BM-MSC-EVs prevented CKD development in surviving mice subjected to renal injury induced by treatment with lethal dose of cisplatin (Bruno et al., 2012). The strategy of multiple administration of BM-MSC-EVs was also employed in a murine model of remnant kidney-induced CKD, characterized by glomerular sclerosis and interstitial fibrosis. In this CKD model, BM-MSC-EV treatments improved renal function and reduced tubular injury, interstitial lymphocyte infiltration and collagen deposition (He et al., 2012).

### 3.2 Possible mechanisms of action of BM-MSC-EVs

More recent published research has shown that BM-MSC-EVs can modulate key pathways involved in CKD development, including inflammation, fibrosis and oxidative stress and the observed beneficial effects were correlated with EV-cargo, in particular with proteins and RNAs (Table 1).

**TABLE 1:** Effects of MSC-EVs in different CKD models.

Sources	*In vivo* model of CKD	EV cargo	Biological effects	References
BM-MSCs	Rat model of UUO	MFG-E8	Regulation of RhoA/ROCK pathway	Shi et al. (2020)
	Mouse model of UUO	miR-29, miR-30	Regulation of EMT process	He et al. (2015)
	Mouse model of UUO	miR-21a-5p	- Reduction of phosphofructokinase muscle isoform - Attenuation of glycolysis	Xu et al. (2022)
	Mouse model of DN	let-7, miR-30, miRNA-29, miRNA-24, miRNA-21	- Amelioration of glomerular and interstitial fibrosis - Downregulation of pro-fibrotic markers	Grange et al. (2019)
	Mouse model of AAN	-	- Reduction of renal dysfunction - Reduction of tubular necrosis - Reduction of interstitial fibrosis - Downregulation of pro-fibrotic markers	Kholia et al. (2020)
	Rat model of 5/6 nephrectomy	-	- Upregulation of Klotho activity and expression - Amelioration of renal pathological features	Wan et al. (2022)
UC-MSCs	Rat model of UUO	Proteins of the ubiquitination/degradation proteome	- Inhibition of YAP activity - Amelioration of renal function - Reduction of interstitial fibrosis - Downregulation of pro-fibrotic proteins	Ji et al. (2020)
	Mouse model of UUO	miR-874-3p	- Reduction of renal tubular epithelial cell injury - Inhibition of necroptosis - Restoration of mitochondrial homeostasis	Yu et al. (2023)
	Mouse model of DN	miR-22-3p	- Reduction of pro-inflammatory protein expression - Reduction of NLRP3 activation	Wang C. et al. (2023)
	Mouse model of DN	miR-424-5p	- Inhibition of apoptosis - Inhibition of EMT process via YAP1	Cui et al. (2022)
ASCs	Porcine model of MetS and renal artery stenosis	IL-10	- Amelioration of renal inflammation - Reduction of M1-macrophages infiltration - Stimulation of M2-macrophage differentiation	Eirin et al. (2017)
	Swine model of MetS and renovascular disease	Pro-angiogenic mRNAs and proteins	- Reduction of microvasculature loss and remodeling - Upregulation of pro-angiogenic factor expression - Reduction of renal endothelial cell apoptosis - Reduction of oxidative stress	Eirin et al. (2018)
	Rat model of DN	miR-125a	- Reduction of mesangial hyperplasia - Reduction of renal fibrosis	Hao et al. (2021)
	Mouse model of DN	miR-486	- Reduction of mTOR signaling via Smad1 inhibition - Reduction of serum levels of renal dysfunction - Enhancement of autophagy flux - Reduction of podocyte injury	Jin et al. (2019)
	Mouse model of DOCA-salt hypertensive	miR-200	- Reduction of macrophage recruitment - Downregulation of TGFβ signaling - Reduction of pro-fibrotic markers - Prevention of cardiac fibrosis	Lindoso et al. (2020)
HLSCs	Mouse model of 5/6 nephrectomy	MMP-1	- Amelioration of renal function and morphology - Reduction of capillary rarefaction - Amelioration of cardiac function	Ceccotti et al. (2024)
	Mouse model of DN	miR-146, miRNA-29a, let-7, miRNA-30a, miRNA-24, miRNA-21	- Downregulation of pro-fibrotic genes and proteins - Modulation of TGFβ, EGFR, PDGFR and VEGF signaling pathways	Grange et al. (2019)
	Mouse model of AAN	-	- Amelioration of renal function and morphology - Downregulation of pro-fibrotic and pro-inflammatory genes - Reduction of renal infiltration of inflammatory cells	Kholia et al. (2018)
	Mouse model of IRI AKI-CKD	-	- Amelioration of renal function and morphology - Modulation of pro-fibrotic, pro-inflammatory and EMT-genes - Reduction of renal infiltration of inflammatory cells	Bruno et al. (2022)
iPSCs	Mouse model of FA-induced AKI-to-CKD	-	- Amelioration of renal function - Reduction of interstitial fibrosis - Amelioration of capillary rarefaction - Reduction of cell death	Kim et al. (2025)

BM, bone marrow; MFG-E8, milk fat globule–epidermal growth factor–factor 8; EMT, epithelial mesenchymal transition; DN, diabetic nephropathy; UUO, unilateral ureteral obstruction; miR, micro RNA; AAN, aristolochic acid nephropathy; UC, umbilical cord; YAP, Yes-associated protein; NLRP3, nucleotide-binding domain-like receptor 3; ASC, Adipose tissue-derived mesenchymal stem cells; MetS, metabolic syndrome; DOCA, deoxycorticosterone acetate; IRI, ischemia reperfusion injury; EGFR, epidermal growth factor receptor; PDGFR, platelet-derived growth factor receptor; VEGF, vascular endothelial growth factor; TGFb, transforming growth factor-beta; iPSCs, induced pluripotent stem cells; FA, folic acid; AKI, acute kidney injury; CKD, chronic kidney disease.

In a rat model of UUO-induced CKD it has been showed that a single dose of BM-MSC-EVs decreased collagen deposition and protected the microvasculature. The molecular mechanism of action has been investigated, and it has been demonstrated the presence of the milk fat globule–epidermal growth factor–factor 8 (MFG-E8), in EV bioactive cargo. The biological effects of BM-MSC-EVs in UUO-CKD model were reduced by silencing MFG-E8, suggesting its involvement in EV therapeutic activity. In addition, *in vitro* studies indicated that MFG-E8 participates to the regulation of RhoA/ROCK signalling pathway, which in turn is induced by TGFβ1 (Shi et al., 2020). In UUO-induced CKD model it was also demonstrated the involvement of miRNAs shuttled by BM-MSC-EVs in their anti-fibrotic effects. Indeed, BM-MSC-EVs contain specific patterns of miRNAs, such as miR-29 and miR-30 that can modulate CKD development by regulating EMT process. Moreover, BM-MSC-EV treatment induced an amelioration of renal function and morphology with a concomitant reduction in the expression of alpha smooth muscle actin (alpha-SMA) and an increase in the expression of E-cadherin (He et al., 2015). Furthermore, BM-MSC-EVs are highly enriched in miR-21a-5p. It has been reported that knockdown of this miRNA reduced the renoprotective effect of BM-MSC-EVs in UUO-model of CKD. Mechanistically, the expression of phosphofructokinase muscle isoform, a rate-limiting enzyme of glycolysis, is repressed by miR-21a-5p, thereby attenuating glycolysis in tubular cells (Xu et al., 2022).

The relevant role of miRNAs shuttled by BM-MSC-EVs for their anti-fibrotic activity was evaluated also in a model of CKD development induced by diabetic nephropathy (DN). The administration of multiple doses of BM-MSC-EVs in DN mice prevented the progression of CKD, ameliorating interstitial and glomerular fibrosis. EV-treated mice showed not only functional and histological amelioration, but also a downregulation of pro-fibrotic markers, such as Collagen I, alpha-SMA and TGFβ. The miRNA cargos of BM-MSC-EVs were characterized and bioinformatic analyses indicated that they could act on common targets such as pro-fibrotic pathways (TGFβ, insulin growth factor 1, platelet derived growth factor), which is consistent with the observed *in vivo* anti-fibrotic effect (Grange et al., 2019).

Moreover, BM-MSC-EVs showed therapeutic effects in the CKD model of aristolochic acid nephropathy (AAN). Treatment with multiple doses of MSC-EVs induced a significant reduction in plasmatic markers of renal dysfunction, tubular necrosis, and interstitial fibrosis. Furthermore, infiltrate of CD45^+^ positive immune cells, fibroblasts, and pericytes were significantly reduced in renal parenchyma of AAN-mice treated with BM-MSC-EVs. Molecular analyses showed that BM-MSC-EVs significantly reduced AA-mediated induction of pro-fibrotic genes, such as alpha-SMA, Collagen I and TGFβ (Kholia et al., 2020).

BM-MSC-EVs ameliorated renal pathological features also in a model of partial nephrectomy, upregulating the expression and the activity of Klotho (Wan et al., 2022). The regulation of Klotho expression may be a possible strategy for CKD treatment that can be achieved by BM-MSC-EV administration.

It has been reported that BM-MSC-EVs can attenuate *in vitro* and *in vivo* mitochondrial damage and inflammation (Mao et al., 2022). In particular, BM-MSC-EVs can ameliorate IRI increasing the expression of mitochondrial transcription factor A (TFAM) and of mitochondria-related gene, and ATP production in the kidneys (Zhao et al., 2021). These effects were, at least in part, due to TFAM mRNA and mtDNA shuttling by MSC-EVs, since EVs obtained from TFAM-knockdown MSCs, showed limited abilities to rescue the TFAM deficiency and mitochondrial damage (Zhao et al., 2021).

### 3.3 Other possible MSC-sources of EVs for CKD treatment and their mechanisms of action

Alternative sources of MSC-EVs have been demonstrated to be effective in different CKD pre-clinical models (Table 1).

The abundant and low immunogenicity make human UC-MSCs an appropriate option for EV production. In UUO-induced CKD model, administration of multiple doses of UC-MSC-EVs ameliorated renal function and reduced interstitial fibrosis and related proteins, such as Collagen I, alpha-SMA and TGFβ. To elucidate the mechanism by which UC-MSC-EVs inhibited fibrosis development, the protein profile of this EV population was analysed by LC-MS/MS and was found that the proteins related to ubiquitination/degradation proteome system were enriched in UC-MSC-EVs. These proteins contributed to the inhibition of Yes-associated protein (YAP) activity, leading to the reduction of renal fibrosis development. YAP protein is a co-factor of the Hippo pathway, and its dysregulation may contribute to the progression of renal fibrosis by increasing the deposition of ECM components (Ji et al., 2020). UC-MSC-EV administration in UUO-CKD model reduced the extent of renal tubular epithelial cell injury and promoted post-injury repair also restoring mitochondrial homeostasis and by inhibiting necroptosis. In particular, it has been reported that UC-MSC-EVs contain miR-874-3p that can be involved in regulating programmed necrosis and mitochondrial division (Yu et al., 2023). Furthermore, it has been demonstrated that the protective effect of UC-MSC-EVs in CKD is also due to the regulation of inflammation and immunity. UC-MSC-EVs administration in DN mice, in fact, reduced inflammation, including the expression of different pro-inflammatory cytokines, and the activation of nucleotide-binding domain-like receptor 3 (NLRP3), ameliorating kidney injury. NLRP3 is an important multi-protein complex component of innate immunity and is a target of miR-22-3p, which is a relatively highly expressed miRNA in UC-MSC-EVs. Knocking down miR-22-3p in UC-MSC-EVs reduced their anti-inflammatory activity and beneficial effect in DN mice (Wang Y. et al., 2023). It has also been reported that miR-424-5p carried by UC-MSC-EVs exerts a possible role in the beneficial effects of this EV population in DN. In fact, it could inhibit apoptosis and EMT by targeting YAP1 in renal proximal tubular epithelial cells (Cui et al., 2022).

Among the possible different sources, adipose tissue is an easy collectable source of MSCs. Adipose-derived mesenchymal cells (ASCs) display immunosuppressive properties, low immunogenicity and secrete paracrine factors and ASC-derived EVs that can support tissue regeneration (Marjorie et al., 2018). Interestingly, *Erin A. and colleagues* demonstrated the therapeutic properties of ASC-EVs in a porcine model of Metabolic syndrome (MetS) and renal artery stenosis. MetS is a cluster of cardiovascular disease-related risk factors that is frequently associated with CKD and increases its progression toward ESRD. In this model, a single intrarenal delivery of autologous ASC-EVs exhibited multiple functions. Indeed, EV treatment ameliorated renal inflammation, by reducing infiltrates of pro-inflammatory M1-macrophages and increasing reparative-M2 macrophage differentiation, and improved medullary oxygenation and fibrosis. These renoprotective effects were reduced in pigs treated with IL10-depleted ASC-EVs, where glomerular sclerosis and hypoxia were not attenuated (Eirin et al., 2017). In a swine model of MetS and renovascular disease, a single intra-renal infusion of autologous ASC-EVs restored organ microcirculation. In fact, in stenotic kidney, EV treatment reduced the loss and remodeling of microvasculature and increased the expression of pro-angiogenic factors, such as vascular endothelial growth factor (VEGF), Notch and delta-like ligand 4. Moreover, ASC-EVs reduced both renal endothelial cell apoptosis and oxidative stress by decreasing endothelial cells caspase 3 and the fluorescent staining for the superoxide anion. mRNA sequencing and proteomic analysis highlighted the presence of pro-angiogenic mRNAs (VEGF-A, VEGF receptor) and proteins (VEGF, Hepatocyte Growth Factor) in ASC-EV cargo, which could be correlated to their pro-angiogenic activity (Eirin et al., 2018).

EVs isolated from ASCs also inhibited DN progression by suppressing mesangial hyperplasia and kidney fibrosis. The researchers found that miR-125a was partially responsible for their protective effects (Hao et al., 2021). Moreover, ASC-EVs attenuated spontaneous DN by reducing levels of urine protein, serum creatinine, blood urea nitrogen and podocyte apoptosis. In terms of mechanism, ASCs-EVs enhanced autophagy flux and reduced podocyte injury by inhibiting the activation of mTOR signalling. miR-486 seems to be a key factor for the ASC-EV ability to improve DN symptoms, by reducing Smad1, that in turn can inhibit mTOR activation (Jin et al., 2019). Multiple administrations of EVs isolated from ASCs in deoxycorticosterone acetate (DOCA)-salt hypertensive murine model protected renal tissue structure and function. ASC-EV treatments reduced the recruitment of macrophages in the kidney resulting in the downregulation of specific pro-inflammatory molecules. By performing the analysis of miRNA profile of renal tissue, a selective miRNA signature was identified. One of the key pathways involved was the axis miR-200-TGFβ, that was significantly downregulated in DOCA-mice and restored after EV treatment, with concomitant reduction of gene expression levels of pro-fibrotic markers. Furthermore, ASC-EVs maintained blood pressure within normal levels and prevented cardiac tissue fibrosis (Lindoso et al., 2020). It has been reported that also an MSC-like population derived from human adult liver, called human liver stem cells (HLSCs), is an alternative source of EVs that can revert renal and cardiac fibrosis in a murine model of partial nephrectomy. Multiple administrations of HLSC-EVs improved renal function, morphology and renal capillary rarefaction, with concomitant amelioration of cardiac function and significant reduction of cardiac fibrosis (Ceccotti et al., 2024). Proteomic analyses indicated the presence of several proteins involved in IL-10, p53 and PI3K signaling pathways, and of proteases with ECM remodeling activity, that can influence the anti-fibrotic effect of this EV population (Bruno et al., 2020). Pre-treatment with specific anti-matrix metalloproteinase 1 (MMP-1) blocking antibody decreased HLSC-EV *in vivo* anti-fibrotic activity, indicating a possible involvement of MMP-1 in their biological effect. The beneficial effect of HLSC-EVs is influenced not only by their protein cargo, but also by their RNA content. In different murine models of CKD development (DN, AAN, IRI and partial nephrectomy), it has been reported that multiple injections of HLSC-EVs cause the downregulation of pro-fibrotic genes, as Collagen, alpha-SMA, TGFβ, indicating that this EV population may influence the expression of genes involved in key pathways of CKD-progression (Grange et al., 2019; Kholia et al., 2018; Bruno et al., 2022; Ceccotti et al., 2024). Indeed, in the cargo of HLSC-EVs there are several miRNAs that can target genes involved in pro-fibrotic pathways, such as TGFβ, epidermal growth factor and platelet derived growth factor receptors and VEGF (Grange et al., 2019). In the murine model of IRI-induced AKI-to-CKD transition, treatment with HLSC-EVs interfered with CKD development also by reducing both the infiltration of inflammatory cells and the gene expression level of pro-inflammatory cytokines and EMT markers (Bruno et al., 2022). Moreover, the anti-inflammatory effect of HLSC-EVs has also been described in AAN murine model (Kholia et al., 2018).

Another interesting MSC source is represented by induced pluripotent stem cells (iMSCs), which is advantageous over other MSC-sources, since iMSCs can be easily expanded to obtain high numbers of homologous clonally-derived MSCs to avoid cell heterogenicity (Lee et al., 2023). EVs obtained by iMSCs exerted a protective effect on AKI-to-CKD transition, induced by folic acid administration. Repeated administration of iMSC-EVs ameliorated renal function, reduced interstitial fibrosis, inflammation, cell death and immune cells infiltrating in the kidneys. Furthermore, EV administration reversed capillary rarefaction in renal tissue, indicating that iMSC-EVs have the potential to block AKI-to-CKD transition (Kim et al., 2025). However, it has not yet been determined the mechanism of action of iMSC-EVs to inhibit AKI-to-CKD transition and further studies will be necessary.

## 4 Concluding remarks and future perspectives

Several published studies suggest that MSC-EVs can effectively ameliorate renal function and morphology, providing evidence of their multi-level nephroprotection capabilities. Treatment of main causes of ESRD, such as diabetic nephropathy and hypertension, could be envisaged based on growing evidence of the beneficial effects of MSC-EVs in preclinical animal models (Cao et al., 2022). Indeed, MSC-EVs they have proved effective in reducing proteinuria and serum creatinine and even improving renal pathology. Immunomodulatory effects of MSC-EVs could provide an additional rationale to treat CKD secondary to glomerulonephritis which may still include an active inflammatory component, such as lupus nephritis (Quaglia et al., 2022) and combination of MSCs and immunosuppressants may have synergistic effects in refractory forms (Liu et al., 2023).

Despite the high number of experimental studies that demonstrated the efficacy of MSC-EVs in different pre-clinical models of CKD, only one study showed data obtained in human patients. Nassar et al., 2016, reported the use of EVs derived from UC-MSCs in patients with stage III or IV CKD. The patients received two doses of 100 μg of EVs/Kg body weight in two consecutive weeks. The first dose was intravenously administered; the second dose was intra-arterially administered. The control placebo group received saline infusion. The EV-treated patients (n = 20) achieved an improvement of GFR, creatinine serum level, blood urea and an amelioration of urinary albumin/creatinine ratio, for 1 year study period. The EV-treatments reduced the levels of circulating pro-inflammatory cytokines and increased the levels of anti-inflammatory cytokines, such as IL-10. Renal biopsy performed 3 months after EV-treatments revealed that renal tissues upregulated the expression of markers of cell regeneration and differentiation. Notable, patients that received EVs did not experience any significant adverse effects during or after treatments. Hence, this study indicated that MSC-EV therapy is safe and could improve inflammation and renal function in CKD patients, as reported in pre-clinical animal models of CKD.

The lack of new clinical trials in humans could be due to the absence of consensus on isolation method and characterisation protocols of MSC-EVs. MSC-EVs can offer superior advantages as therapeutics over the use of the cells of origin, due to their acellular nature and small size, higher safety profile, lower immunogenicity and not pro-tumorigenic effect *in vivo*. For the translation of MSC-EV treatments to clinical practice, numerous manufacturing issues have to be addressed. In particular, it will be necessary to standardize the isolation method, to reach the scalability of the process, to obtain uniformity among different batches and to set up the potency assays, all these aspects should be adherent to current Good Manufacturing Practices. Moreover, additional studies will be necessary to identify the best cell sources for EV production and to find the optimal administration regimen in terms of dose and mode of injection.

Drugs commonly used to treat CKD patients have a spectrum of pleiotropic actions (Pantelidis et al., 2018) which partly overlap with those of MSC-EVs. SGLT-2i have anti-oxidant, anti-inflammatory, anti-fibrotic and anti-apoptotic effects which play an important role in cardio-nephroprotection and account for the impressive clinical impact that have shown even in moderate-risk CKD patients (Afsar and Afsar, 2023; Sloan, 2024). Furthermore, SGLT2i have anti-ageing effects on the vasculature probably through activation of Nrf-2 and soluble Klotho (Maltese et al., 2023), two pivotal systems involved in cellular senescence in CKD. Similarly, administration of BM-MSC-EVs in a rat model of 5/6 nephrectomy-induced CKD significantly upregulated the expression of Klotho, resulting in reduced 24 h urinary protein excretion and improved renal function and histology (Wan et al., 2022). It has been shown that UC-MSC-EVs can alleviate age-related degenerative disorders in vasculature and many organs including kidney, heart, muscle and brain (Zhang et al., 2024) and they even hold promise as potential treatment for Alzheimer’s disease (Rather et al., 2023).

Although the important shared actions between drugs and EVs may represent a potential prospect for a combinatorial therapy, only a few studies discussed about the possible synergy between MSC-EVs and drugs. In a rat model of hypertensive CKD, combination of the recombinant form of the endogenous human relaxin-2 peptide hormone, known as serelaxin (RLX), with BM-MSC-EVs determined a better nephroprotective effect than RLX alone (Li et al., 2021). Furthermore, combined administration of angiotensin 2 receptor blocker Losartan with ASC-EVs has been recently demonstrated to promote additional nephroprotection in a partial nephrectomy CKD model, as compared to treatment with either Losartan or EVs alone. A synergic improvement was shown at histological level (reduction of glomerulosclerosis and of interstitial inflammatory infiltrates), on blood pressure control and on anti-proteinuric effect (Noda et al., 2025). As RAS blockers represent a pivotal treatment of CKD and exert anti-inflammatory and anti-fibrotic effects (Mirabito Colafella et al., 2019), co-administration of MSC-EVs could represent a novel approach to enhance these properties.

Furthermore, MSC-EVs could be integrated into current therapies aimed at reducing the burden of cardiovascular disease (Neves et al., 2023), which is a hallmark of CKD in affected patients and a prominent contributor to morbidity and mortality (Pohlman et al., 2025). MSC-EVs can interfere with important mechanisms of vascular damage, such as endothelial-mesenchymal transformation (Sishuai et al., 2024) and vascular calcifications (Luo et al., 2022). Furthermore, MSC-EVs may decrease cardiac fibrosis after myocardial infarction and inhibit endothelial cell apoptosis in stroke, suggesting potential application to treat sequelae of major vascular events (Neves et al., 2023). Similarly, another important setting could be the AKI-CKD progression, as MSC-EVs can slow or even reverse progression of acute to chronic lesions (Birtwistle et al., 2021) by stimulating tissue reparative mechanisms (Wang et al., 2025).

Although future studies are necessary to establish the best MSC-EV source, dose, administration route and techniques in GMP condition to obtain reproducible MSC-EV-batches, published results underline the possibility to use MSC-EV administration as a potential adjuvant in CKD treatment. Moreover, this could be a good opportunity to faster clinical application of MSC-EVs.
